# Interplay Between BALL and CREB Binding Protein Maintains H3K27 Acetylation on Active Genes in *Drosophila*

**DOI:** 10.3389/fcell.2021.740866

**Published:** 2021-09-28

**Authors:** Ammad Shaukat, Muhammad Haider Farooq Khan, Hina Ahmad, Zain Umer, Muhammad Tariq

**Affiliations:** Epigenetics and Gene Regulation Laboratory, Department of Biology, Syed Babar Ali School of Science and Engineering, Lahore University of Management Sciences, Lahore, Pakistan

**Keywords:** CBP, epigenetics, histone modifications, gene activation, ChIP-seq

## Abstract

CREB binding protein (CBP) is a multifunctional transcriptional co-activator that interacts with a variety of transcription factors and acts as a histone acetyltransferase. In *Drosophila*, CBP mediated acetylation of histone H3 lysine 27 (H3K27ac) is a known hallmark of gene activation regulated by trithorax group proteins (trxG). Recently, we have shown that a histone kinase Ballchen (BALL) substantially co-localizes with H3K27ac at trxG target loci and is required to maintain gene activation in *Drosophila*. Here, we report a previously unknown interaction between BALL and CBP, which positively regulates H3K27ac. Analysis of genome-wide binding profile of BALL and CBP reveals major overlap and their co-localization at actively transcribed genes. We show that BALL biochemically interacts with CBP and depletion of BALL results in drastic reduction in H3K27ac. Together, these results demonstrate a previously unknown synergy between BALL and CBP and reveals a potentially new pathway required to maintain gene activation during development.

## Introduction

In metazoans, covalent modifications of histones act in a combinatorial manner to regulate developmental gene expression (Allis and Jenuwein, 2016; Zhao and Shilatifard, 2019). Among these modifications, histone H3 lysine 27 acetylation (H3K27ac) is considered a hallmark of trithorax group (trxG) mediated gene activation that antagonizes repression by Polycomb group (PcG) proteins (Tie et al., 2009). Although the anti-silencing effect of H3K27ac and its catalyzing enzyme CREB binding protein (CBP) is widely documented in flies and mammals, how this CBP mediated H3K27ac is maintained on actively transcribed genes remains elusive (Tie et al., 2009; Pasini et al., 2010). In *Drosophila*, presence of only one homolog of CBP known as *nejire*, makes it suitable to understand its function. Importantly, chromatin immunoprecipitation sequencing (ChIP-seq) data from previously published reports revealed the presence of CBP on a majority of actively transcribed genes in *Drosophila* S2 cells (Philip et al., 2015). Our lab recently showed that *Drosophila* Ballchen (BALL), a known serine-threonine kinase that mediates histone H2AT119 phosphorylation (Aihara et al., 2004), associates with trxG target genes enriched with H3K27ac (Khan et al., 2021). In this report, we have further analyzed the link between BALL, H3K27ac and CBP. We have discovered that BALL not only binds to chromatin enriched with H3K27ac but also shares more than 77% genomic binding sites with CBP. Both BALL and CBP primarily associate with transcription start sites (TSS) and regions up to 1 Kb upstream of TSS. Four of top five DNA motifs in chromatin that CBP binds are also bound by BALL. Importantly, BALL biochemically interacts with CBP and positively contributes to maintenance of H3K27ac by CBP since depletion of BALL leads to diminished H3K27ac.

## Results and Discussion

**FIGURE 1:**
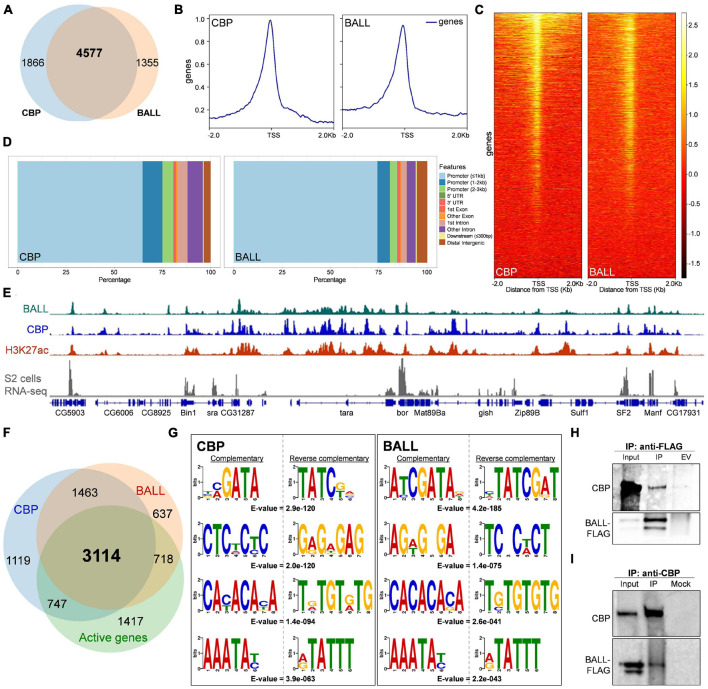
Genome-wide occupancy of BALL, CBP, and their biochemical interaction in *Drosophila* S2 cells. **(A)** Venn diagram depicting substantial overlap between CBP and BALL bound genes in S2 cells. Both BALL and CBP are co-bound at 4577 genes. **(B,C)** Comparison of genome-wide occupancy of CBP and BALL reveals an enormous similarity. Both CBP and BALL preferentially bind chromatin upstream to the TSS **(B)**. Heat maps covering ± 2 Kb region from TSS illustrate CBP and BALL occupancy across the genome **(C)**. CBP and BALL mainly co-occupy the regions from TSS to 1 Kb upstream. **(D)** Percentage distribution of CBP and BALL binding across different genome features shows a similar pattern where both CBP and BALL mainly reside in promoter regions. **(E)** Integrated genome browser view of the ChIP-seq data shows overlap between chromatin binding profiles of BALL (top panel), CBP (middle panel) and H3K27ac (lower panel). Genes co-occupied by BALL and CBP display active transcription as inferred by RNA-seq analysis of S2 cells (bottom panel). **(F)** Venn diagram depicting CBP and BALL enriched genes overlaid with actively transcribed genes in S2 cells. Majority of the genes that were co-bound by CBP and BALL were found to be transcriptionally active. **(G)** Four of the top five short DNA binding motifs within CBP associated chromatin, generated from MEME-ChIP database, show-striking similarity with the similar motifs within BALL bound chromatin. Each motif is presented with its respective reverse complementary motif and *E*-value. **(H)** Immunoblots of endogenous CBP and FLAG-tagged BALL after co-immunoprecipitation (IP) using anti-FLAG antibody in *Drosophila* S2 cells containing inducible BALL-FLAG. As compared to IP from empty vector (EV) control cells, noticeable enrichment of CBP can be seen in IP using anti-FLAG antibody, which also resulted in enrichment of FLAG-BALL. **(I)** Immunoblots of endogenous CBP and FLAG-tagged BALL after co-immunoprecipitation (IP) using anti-CBP antibody in *Drosophila* S2 cells. BALL-FLAG is enriched after IP with anti-CBP antibody as compared to mock IP used as negative control. Enrichment of CBP validates successful IP.

Recently we reported that BALL exhibits trxG like behavior by contributing to the maintenance of gene activation in flies (Khan et al., 2021). Since BALL was shown to co-localize with Trithorax (TRX) at genes enriched with H3K27ac, we investigated if BALL binds to chromatin together with CBP. To this end, we analyzed ChIP-seq data of BALL (Khan et al., 2021) and CBP (Philip et al., 2015) from *Drosophila* S2 cells. Comparison of BALL and CBP binding to chromatin revealed their co-occupancy at 4577 genes (Figure 1A and Supplementary Table 1). Further analysis revealed that both BALL and CBP shared binding sites in genomic regions up to 1 Kb upstream of the transcription start sites (TSS), however, CBP is also found at the regions that are further upstream of TSS (Figures 1B,C). This finding is in line with the reported binding of CBP in promoter as well as enhancer regions (Philip et al., 2015; Boija et al., 2017). Analysis of the genomic distribution of CBP and BALL in terms of their binding at promoter regions, gene body, introns and intergenic regions also revealed similar chromatin binding patterns (Figure 1D). Of 6,553 overlapping binding sites for both BALL and CBP, 5,865 were mainly clustered on promoter regions (Supplementary Figure 1 and Supplementary Table 2). Importantly, promoters of different genes have variable number of peaks, i.e., some promoters have multiple binding sites while others have a single binding site. Binding profile of BALL closely mirrored that of CBP and H3K27ac at a majority of genomic regions. Additionally, most of these shared loci were also expressed in S2 cells, as highlighted by analysis of RNA-seq data (Gan et al., 2010; Figure 1E). Of 4577 genes where both BALL and CBP are present, 3114 (68%) were indeed found to be actively transcribed in S2 cells (Figure 1F and Supplementary Table 1). Comparison between top scoring DNA motifs present within chromatin regions where both BALL and CBP preferentially bind revealed a distinct similarity (Figure 1G). Collectively, our data highlights that BALL binding pattern substantially overlaps the binding of CBP across the whole genome. These findings prompted us to investigate whether BALL and CBP biochemically interact with each other. To this end, co-immunoprecipitation (co-IP) experiments were performed using S2 cells that stably expressed FLAG-tagged BALL. Immunoprecipitation of BALL-FLAG resulted in precipitation of endogenous CBP as compared to empty vector control (Figure 1H). To confirm this interaction, a reciprocal co-IP was performed using anti-CBP antibody, which resulted in immunoprecipitation of BALL-FLAG (Figure 1I) as compared to mock IP. These results illustrate a hitherto unknown interaction between BALL and CBP, which may explain their co-occupancy at actively transcribed regions.

**FIGURE 2:**
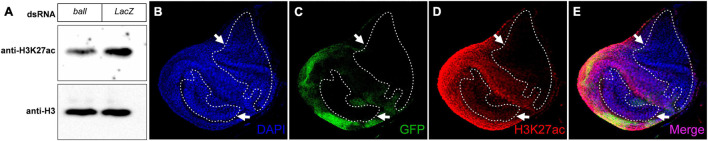
Depletion of BALL affects H3K27ac levels. **(A)** Western blot analysis of whole cells lysates from *Drosophila* S2 cells treated with dsRNA against *ball* exhibit reduced H3K27ac. Cells treated with dsRNA against *LacZ* served as control while total levels of histone H3 were used as a normalization control in immunoblot. Analysis of the relative intensities of H3K27ac signal, normalized against total H3 levels using ImageJ, showed more than 37% reduction in H3K27ac upon *ball* depletion. Experiment was performed thrice and replicates are shown in Supplementary Figure 2. **(B–E)** Haltere imaginal discs containing mitotic clones of *ball*^2^ were stained with anti-H3K27ac **(D)**. Mitotic clones, marked by the absence of GFP, showed drastic reduction of H3K27ac. Imaginal discs showed uniform DAPI staining **(B)**. Mitotic clones are encircled and highlighted with arrows. The *ex vivo* knock down of *ball* was performed in triplicate while the mitotic clone experiment was performed in duplicate.

Next, we investigated if depletion of BALL affects CBP mediated H3K27ac levels globally. Western blot analysis of cells where *ball* was knocked down revealed drastic reduction in H3K27ac levels as compared to control cells treated with dsRNA against *LacZ* (Figure 2A). The global levels of histone H3 remained unchanged which were used as normalization control to quantify reduction in levels of H3K27ac (Supplementary Figure 2). To validate this finding *in vivo*, we generated homozygous *ball*^2^ mitotic clones using the flp/FRT system. Immunostaining of imaginal discs with *ball*^2^ mutant clones showed a drastic reduction in H3K27ac (Figures 2B–E and Supplementary Figure 3). Notably, not all homozygous *ball*^2^ mitotic clones show diminished H3K27ac which reiterates similar effects observed in *trx* mutant clones on expression of actively transcribed genes. It could be explained by the complex interplay of different cell signaling pathways in imaginal discs (Klymenko and Müller, 2004).

Together, these results demonstrate that BALL positively contributes to CBP mediated H3K27ac but the molecular nature of this interaction between BALL and CBP remains unclear. Whether they are part of a single multi-protein complex and if they directly interact with each other or it involves an indirect interaction remains elusive. Since BALL is a histone kinase, it is imperative to investigate whether the impact that BALL exerts on H3K27ac is through the phosphorylation of neighboring H3 residues or due to the direct phosphorylation of CBP or its interacting proteins. Interestingly, VRK1 (Vaccinia related kinase 1), the mammalian homolog of BALL, was shown to phosphorylate CREB at serine 133 (Kang et al., 2008). This phosphorylation is known to enhance CREB interaction with CBP, leading to increased transcription of CREB target genes (Radhakrishnan et al., 1998). The VRK1, CBP and CREB nexus may explain the regulation of CBP mediated gene activation by BALL in flies, which warrants further investigation.

## Materials and Methods

### Chromatin Immunoprecipitation Sequencing Analysis

Chromatin immunoprecipitation sequencing (ChIP-seq) analysis was done as described previously (Khan et al., 2021). Briefly, the sequencing datasets were uploaded to the Galaxy web public server (Jalili et al., 2020). Using Bowtie version 2, the ChIP-seq data was mapped to *Drosophila* genome (dm6) (Langmead et al., 2009). Peak calling was performed using MACS version 2 with FDR (*q*-value) of 0.05 as cut-off and read extension of 200 bp (Zhang et al., 2008). List of annotated peaks was extracted from peak file using ChIPseeker (Yu et al., 2015). Comparative heat maps of BALL and CBP were generated using deepTools, (bamCompare, computeMatrix, and plotHeatmap) (Ramírez et al., 2016). To find out overlapping peaks between BALL and CBP, “bedtools Intersect intervals” was utilized in galaxy database. MEME-ChIP - motif discovery, enrichment analysis and clustering on large nucleotide datasets (Galaxy Version 4.11.2 + galaxy1) was utilized to generate DREME (Discriminative Regular Expression Motif Elicitation) output of BALL and CBP ChIP-seq data for *de novo* motif discovery (Jalili et al., 2020). ChIP-seq data of CBP, BALL, and H3K27ac were taken from GSE64464 (Philip et al., 2015), GSE165685 (Khan et al., 2021) and GSE81795 (Rickels et al., 2016), respectively. RNA-seq data of *Drosophila* S2 cells (GSM480160) (Gan et al., 2010) was mapped using TopHat Gapped-read mapper for RNA-seq data (Galaxy Version 2.1.1). Exon read count was calculated using htseq-count to generate list of active genes.

### Generation of BALL Stable Cell Line

To generate stable cell line with inducible expression of FLAG tagged BALL, *w*^1118^ embryos were used to prepare cDNA and amplify *ball* CDS. The *ball* CDS was first cloned in *pENTR-D-TOPO* entry vector (Thermo Fisher Scientific) followed by sub-cloning in *pMTWHF* destination vector, a gift from Paro lab (Kockmann et al., 2013), by setting up LR Clonase reaction following manufacturer’s protocol (Thermo Fisher Scientific). The resulting *pMT-ball-FLAG* vector map is given in the Supplementary Figure 4. *Drosophila* S2 cells were transfected with *pMT-ball-FLAG* using Effectene transfection reagent (Qiagen) and a stable cell line was generated by following the manufacturer’s instructions (Qiagen).

### Co-immunoprecipitation

For co-immunoprecipitation, *Drosophila* S2 cells expressing FLAG-tagged BALL were washed twice with PBS after harvesting them by centrifugation at 3000rpm for 5 min. Cells were suspended in 1 mL lysis buffer containing 140 mM NaCl, 20 mM Tris (7.4 pH), 1 mM EDTA, 0.5% NP40, 10% glycerol, 0.2 mM Na_2_VO_4_, pepstatin 0.5 μg/mL, leupeptin 0.5 μg/mL, aprotinin 0.5 μg/mL and PMSF 1 mM followed by incubation on ice for 30 min. The lysate was centrifuged at 14,000rpm for 15 min and supernatant was added to 40 μL of M2-FLAG agarose beads (Sigma-Aldrich) which were pre-washed with lysis buffer. After 1 h of incubation, beads were washed thrice with lysis buffer, re-suspended in 30 μL Laemmli Loading buffer and heated at 95°C for 5 min. IP samples were loaded on Novex precast Tris-acetate 3–8% gradient gels (Thermo Fisher Scientific). For CBP IP, 1:1 Protein A and G Dyna beads (Thermo Fisher Scientific) mixture was incubated with anti-CBP antibody overnight. Cell lysates were prepared, and incubated for 1 h at 4°C with the beads-antibody complex. After washing thrice with lysis buffer, samples were proceeded for western blotting as described above.

### *Ex vivo* Knock Down

PCR amplification of the templates for dsRNAs preparation was done using T7 promoter (TAATACGACTCACTATAGGGAGA) tailed oligonucleotides as primers. Primers used for *ball* template are ATAGTTCACCACCCAGCCAG and ATCCTGGTCCGCTTTCTTTT for the DRSC amplicon ID DRSC26607 (Ramadan et al., 2007). Primers used for *LacZ* template are GGAAGATCAGGATATGTGG and CTTCATCAGCAGGATATCC. *In vitro* transcription of the templates was performed using MEGAscript^TM^ T7 Transcription Kit according to the manufacturer’s instructions (Ambion). For setting up the knock down experiment, D. Mel-2 cells were treated with 10 μg/mL of respective dsRNA for 4 days and total cell lysates were prepared in Laemmli loading buffer before heating at 95°C for 5 min.

### Generation of Mitotic Clones

The mutant clones for *ball*^2^ were generated using flp-mediated recombination as described previously (Beuchle et al., 2001). The parental flies for obtaining larvae with the genotype (*y^1^ w^∗^ P{ry^+^, hs-FLP}1/w^∗^; P{neoFRT}82B P{Ubi-GFP}83/P{neoFRT}82B e ball^2^)* were gifted by Alf Herzig (Herzig et al., 2014). To obtain larvae with the aforementioned genotype, a cross was setup between female *HsFlp;* + *; P{neoFRT}82B, P{Ubi-GFP}* and male + *;* + *; P{neoFRT}82B, ball^2^/Tb* flies. All flies were reared at 25°C and the detailed crossing scheme for mitotic clones is given in Supplementary Figure 5. Fifty-five hours after egg laying, larvae with the genotype *y^1^ w^∗^ P{ry^+^, hs-FLP}1/w^∗^; P{neoFRT}82B P{Ubi-GFP}83/P{neoFRT}82B e ball^2^* were given a heat shock for 1 h at 38°C. Sixty-five hours after the heat shock, larval carcasses were inverted, fixed and labeled with antibodies against H3K27ac and GFP. Immunostaining of imaginal discs was done as described previously (Xu and Rubin, 1993). Images were acquired using Nikon C2 Confocal Microscope.

### Antibodies

Antibodies used during this study are as following: mouse anti-GFP (Roche, 11814460001, stock concentration: 0.4 mg/mL, diluted 1:50 for immunofluorescence), mouse anti-FLAG M2 (Sigma Aldrich, F3165, stock concentration: 4 mg/mL diluted 1:2,000 for western blotting), rabbit anti-H3K27ac (Abcam, Ab4729, stock concentration: 1 mg/mL, diluted 1:2,000 for western blotting and 1:75 for immunofluorescence), mouse anti-H3 (Abcam, ab10799, stock concentration: 1 mg/mL, diluted 1:10,000 for western blotting), rabbit anti-CBP serum (gift from Alexander M. Mazo, IP: 5 μL, WB: 1:3,000).

## Data Availability Statement

The datasets presented in this study can be found in online repositories. The names of the repository/repositories and accession number(s) can be found in the article/Supplementary Material.

## Author Contributions

AS and MHFK performed the research. MHFK, AS, ZU, HA, and MT wrote the manuscript. All authors contributed to the article and approved the submitted version.

## Conflict of Interest

The authors declare that the research was conducted in the absence of any commercial or financial relationships that could be construed as a potential conflict of interest.

## Publisher’s Note

All claims expressed in this article are solely those of the authors and do not necessarily represent those of their affiliated organizations, or those of the publisher, the editors and the reviewers. Any product that may be evaluated in this article, or claim that may be made by its manufacturer, is not guaranteed or endorsed by the publisher.
