# Supplementary material for: Interplay Between BALL and CREB Binding Protein Maintains H3K27 Acetylation on Active Genes in Drosophila
Source: Front Cell Dev Biol. 2021 Sep 28;9:740866. doi: 10.3389/fcell.2021.740866 (PMC8509297; doi:10.3389/fcell.2021.740866)
Supplement: Supplementary file 3 [file Data_Sheet_1.PDF]

Supplementary Figure 1. Genomic features distribution of BALL and CBP co-occupied regions. ChIPseeker was utilized to generate a pie chart that represents distribution of BALL and CBP co-occupied peaks across genome. BALL and CBP predominantly co-occupies promoter regions that are less than 1kb upstream of TSS (=1 kb), followed by the promoter regions which are 1-2kb and 2-3kb upstream of TSS. Total 89.5% of the BALL and CBP co-occupied regions are in promoters.

Supplementary Figure 2. Depletion of BALL leads to diminished levels of H3K27ac. All three biological replicates for analysis of H3K27ac levels after *ex vivo* BALL depletion using double stranded RNA (dsRNA) as compared to lacZ dsRNA treated cells used as a control. Western blot analysis of whole cells lysates from *Drosophila* S2 cells treated with dsRNA against *ball* exhibit reduced H3K27ac in each of the three replicates. Cells treated with dsRNA against *LacZ* served as control while total histone H3 levels were used for normalization to quantify levels of H3K27ac. Analysis of the relative intensities of H3K27ac signal using ImageJ showed 37.7% average reduction in H3K27ac upon *ball* depletion. Statistical analysis using t-test represented as bar chart showed that the difference is significant with p-value of 0.0003.

Supplementary Figure 3. Expression of H3K27ac in haltere imaginal disc from wild-type larvae.

Supplementary Figure 4. Graphic representation of the vector backbone containing *pMT-ball-HF.* The coding sequence of *ball* is fused in-frame with coding sequences of 8X *His* and 3X *FLAG* epitopes, under copper inducible promoter (*MT*).

Supplementary Figure 5. Scheme of genetic crosses to obtain *ball* flies of desired genotypes to generate somatic clones of *ball*^{2}. Complete crossing scheme that is used to generate larvae with *ball*^{2} homozygous somatic clones is depicted. Green are the fly lines used as primary source while orange are the fly lines generated in this procedure. Blue is the genotype of the larvae that were used to finally generate somatic clones. All crosses were setup at 25°C.
