# Supplementary figures and images for: Interplay Between BALL and CREB Binding Protein Maintains H3K27 Acetylation on Active Genes in Drosophila
Source: Front Cell Dev Biol. 2021 Sep 28;9:740866. doi: 10.3389/fcell.2021.740866 (PMC8509297; doi:10.3389/fcell.2021.740866)

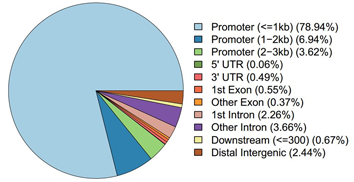

Supplement: Supplementary file 4 [file Image_1.TIF]

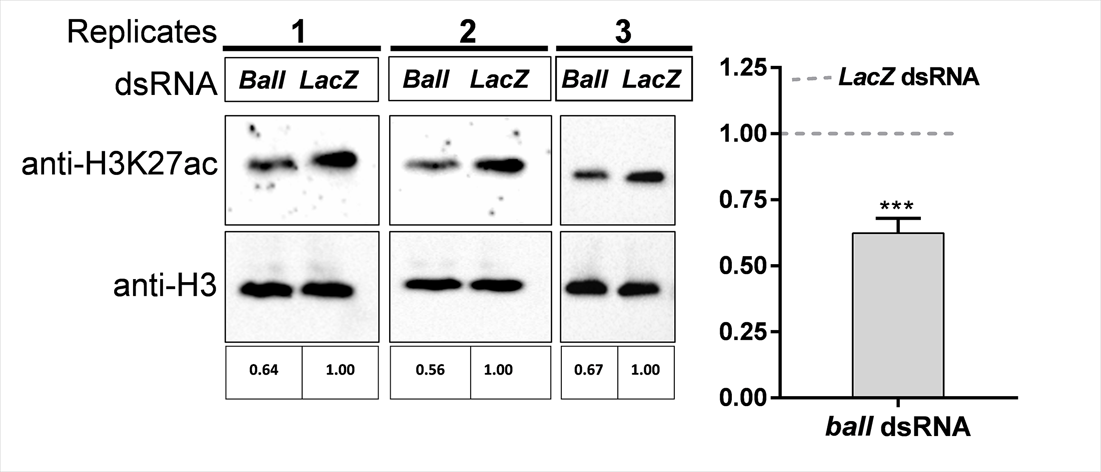

Supplement: Supplementary file 5 [file Image_2.TIF]

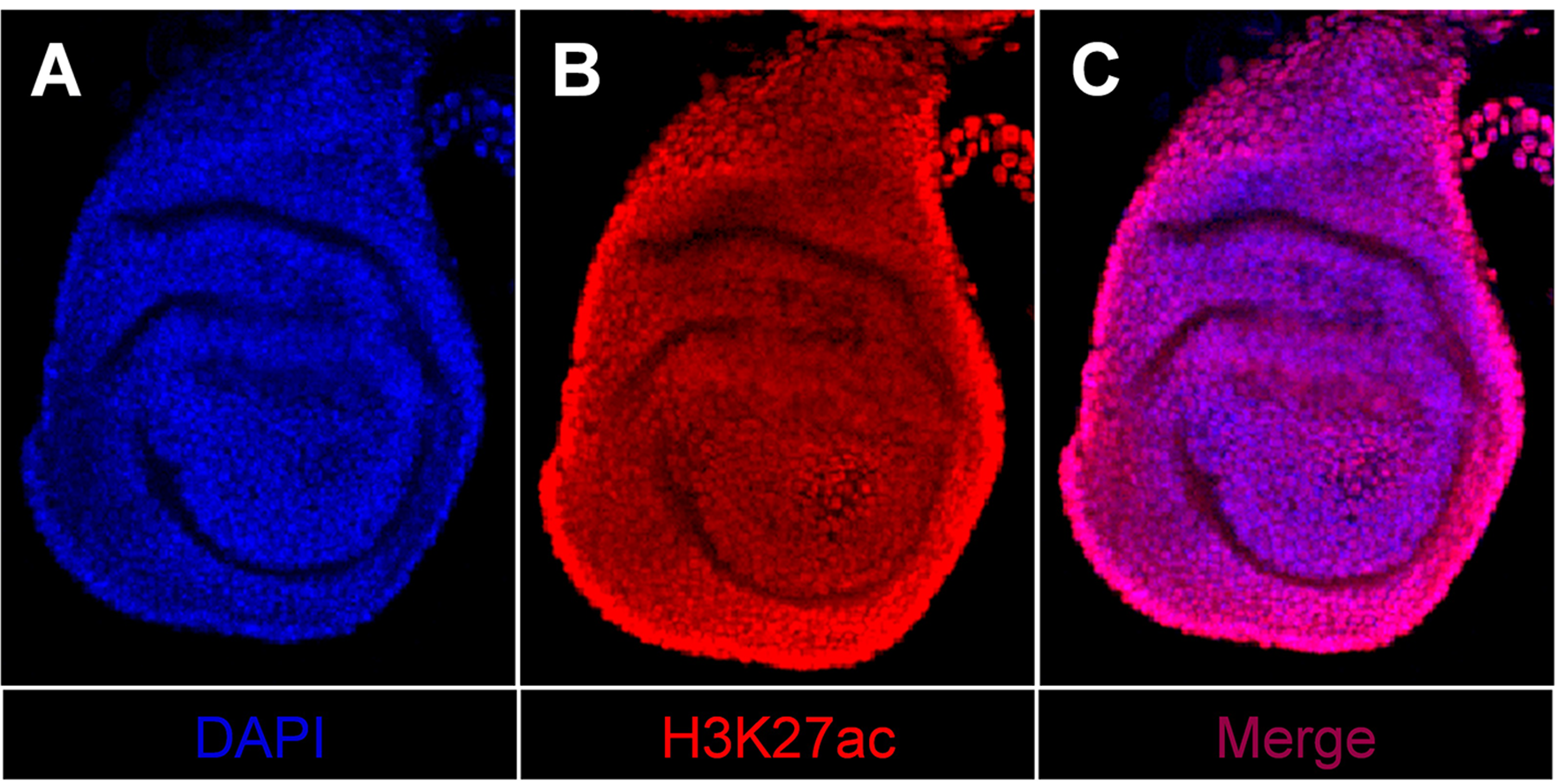

Supplement: Supplementary file 6 [file Image_3.TIF]

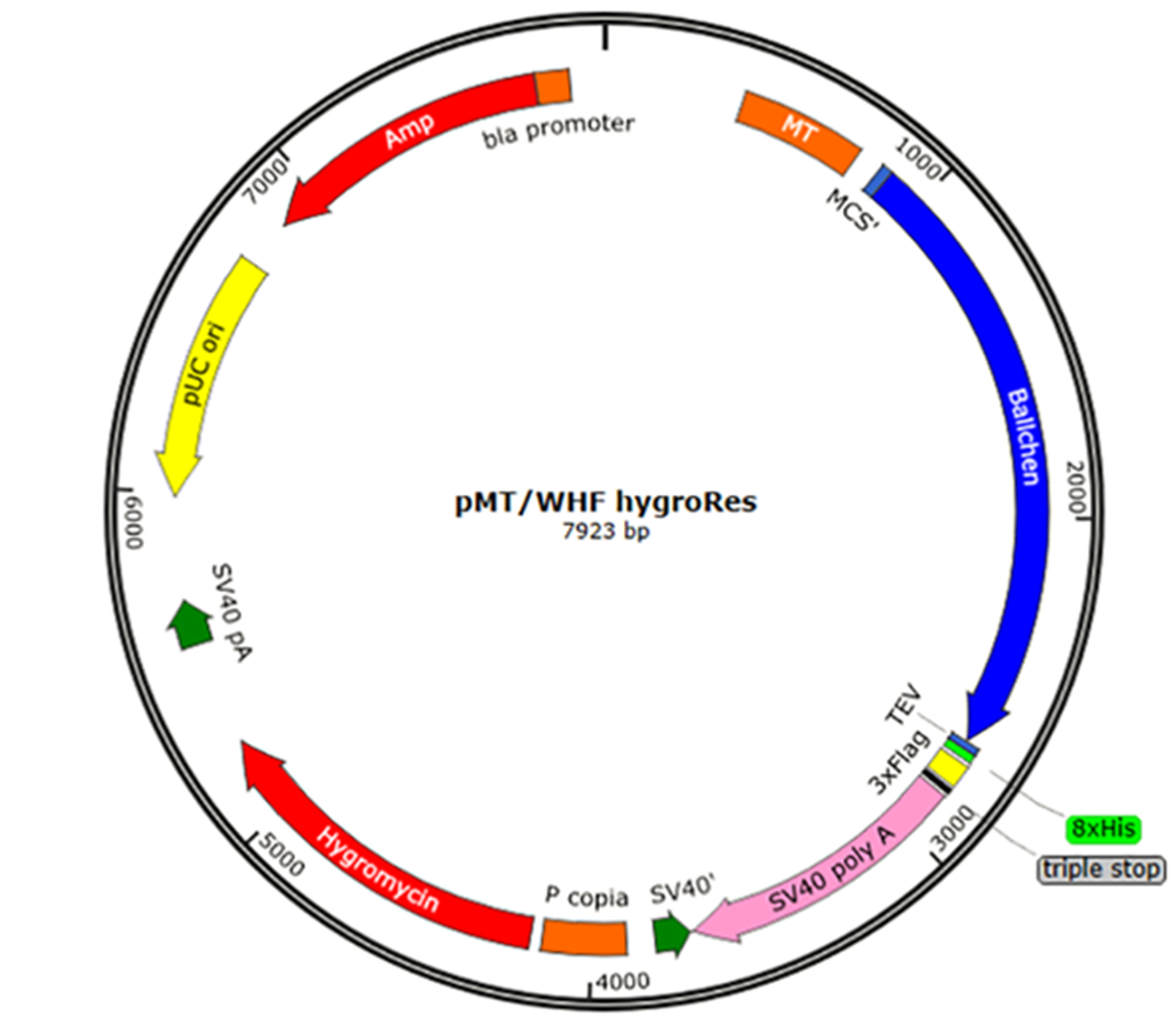

Supplement: Supplementary file 7 [file Image_4.TIF]

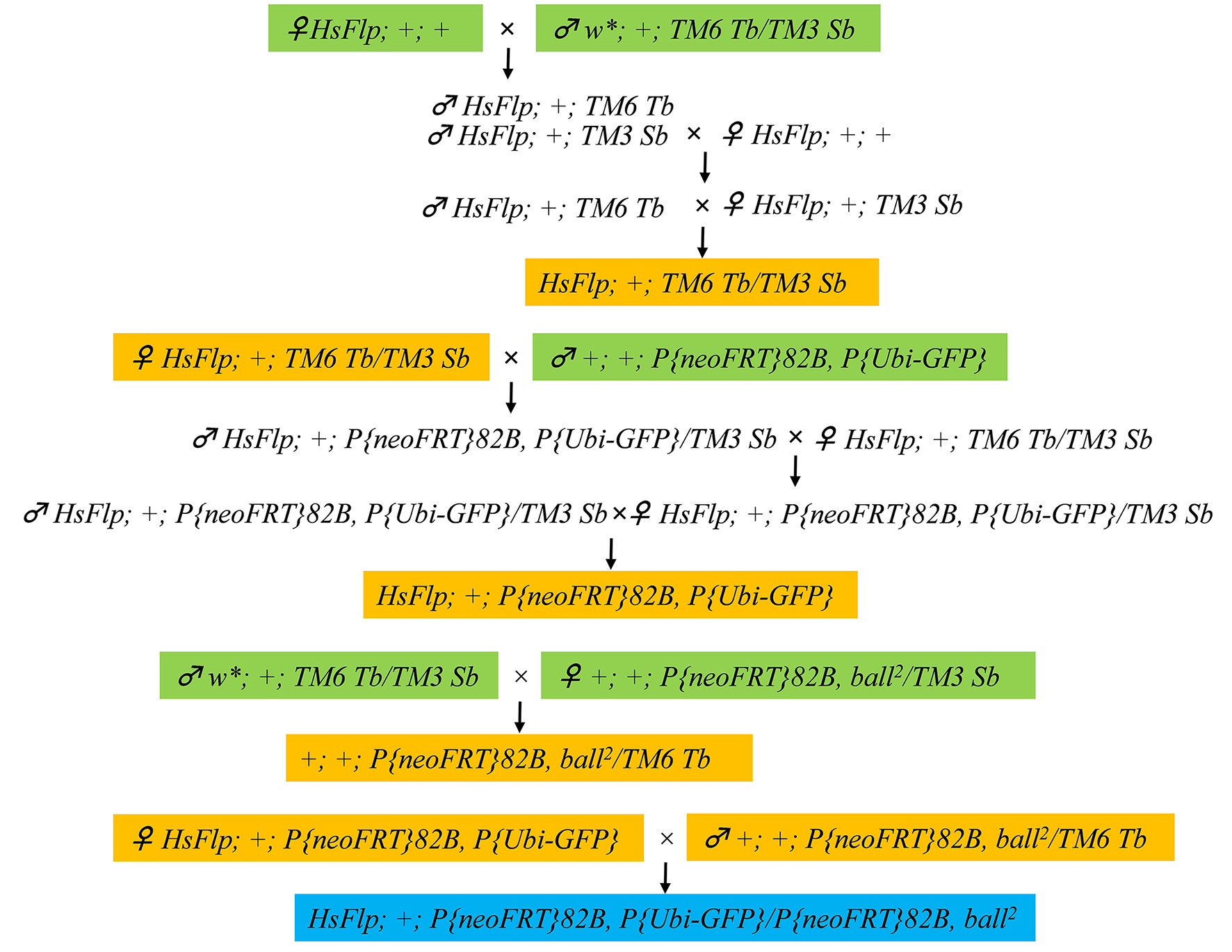

Supplement: Supplementary file 8 [file Image_5.TIF]
